# Supplementary material for: Utilizing natural competence to genetically manipulate Lactobacillus iners
Source: Appl Environ Microbiol. 2026 Aug 10;92(9):e00441-26. doi: 10.1128/aem.00441-26 (PMC13599483; doi:10.1128/aem.00441-26)
Supplement: Supplementary file 1 [file aem.00441-26-s0001.docx]

**Supplemental Material**

**Figure S1. Genomic analysis and detailed identity scores of putative *L. iners* competence operons to known competent model bacteria.** *L. iners* strains from Kenyan sex workers were examined for competence genes that may be homologous to model Gram-positive naturally competent bacteria (*B. subtilis*, *S. thermophilus*, *S. pneumoniae*).

**Figure S2. RefSeq locus tags and homology for Tn*916* integrative and conjugative elements across *L. iners* strains.**

**Figure S3. Overview of transformation protocol for *L. iners*.**

**Figure S4. Restriction-modification systems for *L. iners* strains.** RM systems in *L. iners* genomes were searched for using MicrobeMod; RM operons generally indicate restriction enzyme (RE) with a cognate methylase (MT) and/or specificity (S) enzyme; singletons indicate incomplete RM system. Coloring indicates homologous RM operons between strains. Non-RM genes are colored white.

**Figure S5. Homology of restriction systems between *L. iners* strains.** Percent shared sequenceidentity between enzymes shown in black linker boxes. Non-RM genes and genes not clustered by clinker (using default sequence identity settings) are in grey.

**Figure S6. Confirmation of generation of an NVM076S08 *comGA^-^ iny^-^* double knockout.** Left gel with *comGA* integration primers (expected band size 2663 bp), right gel with *INY* KO integration primers (expected band size 2534 bp). Negative control has no colonies added.

**Table S1. Genome coordinates and RefSeq annotations of competence operons in Gram-positive models and *L. iners* strains.** Coordinates are genomic coordinates for entire operon (e.g., entire comG operon). Locus tag is RefSeq ID for each gene. Gene symbol and product is from RefSeq annotation. Protein ID is GenBank accession code for protein. Feature Start and Feature End indicate genomic coordinates for an individual gene. Feature Type is coding sequence (CDS). Strand indicates +ve or -ve sense (i.e., how operons are organized relative to one another in terms of which DNA strand encodes for operon).

**See attached Excel File**

**Table S2. Primers used.** Overhangs used for Gibson homology regions underlined.

| Name | Strain(s)/  DNA That Primer Set Was Used On | Sequence (5’-3’) | Description | Experiment Used (Refer to Methods) | |
| --- | --- | --- | --- | --- | --- |
| INY KO Upstream F | NVM025S01, NVM076S08, NVM196S18, and NVM210S01 gDNA | GATCAATTTGCTAAAGAAGTAGATTTCT | Amplifies Gibson fragment that flanks left of *ermBL* insert from respective recipient/erythromycin susceptible strain’s gDNA (~1250 bp upstream of INY ORF and ~first 750 bp of INY ORF) | | Gibson assembly of inerolysin knockout PCR products |
| INY KO Upstream R |  | AAAATGTAGGGGGTATTTTTATCACCTGGATTTTCAGGTG |  |  |  |
| INY KO ermBL F | NVM041S27 gDNA (has overhangs with homology to NVM025S01, NVM076S08, NVM196S18, and NVM210S01 INY) | CACCTGAAAATCCAGGTGATAAAAATACCCCCTACATTTTCGT | Amplifies Gibson fragment that contains ermBL insert from NVM041S27’s gDNA with homology to upstream and downstream inerolysin fragments.  Primers have specific overhangs for recipient strains’ inerolysin fragments | | Gibson assembly of inerolysin knockout PCR products |
| INY KO ermBL R | NVM041S27 gDNA (has overhangs with homology to NVM025S01, NVM196S18, and NVM210S01 INY) | GTTACATGTGCATCAAATACACCCTCTATATCGGTTCA |  |  |  |
| INY KO ermBL R | NVM041S27 gDNA (has overhangs with homology to NVM076S08 INY) | GTTACATGTGAGTCAAATACACCCTCTATATCGGTTCA |  |  |  |
| INY KO Downstream F | NVM025S01, NVM196S18, and NVM210S01 gDNA | AATGAACCGATATAGAGGGTGTATTTGATGCACATGTAACAGCTAAGG | Amplifies Gibson fragment that flanks right of ermBL insert from respective recipient/erythromycin susceptible strain’s gDNA (last ~750 bp of INY ORF and ~1250 bp downstream of INY ORF) | | Gibson assembly of inerolysin knockout PCR products |
| INY KO Downstream F | NVM076S08 gDNA | AATGAACCGATATAGAGGGTGTATTTGACTCACATGTAACGGCTAAGG |  |  |  |
| INY KO Downstream R | NVM025S01 and NVM076S08 gDNA | TACGGCAGGTCCTACTGACA |  |  |  |
| INY KO Downstream R | NVM196S18 and NVM210S01 gDNA | TCGCTAAAACCAGACCAATT |  |  |  |
| Final INY KO Assembly F | NVM196S18 and NVM210S01 INY KO Gibson assemblies | GATCAATTTGCTAAAGAAGTAGATTTCT | Primers used to amplify finalized Gibson assemblies  (same as upstream F and downstream R primers for respective recipient strains) | | Gibson assembly of inerolysin knockout PCR products |
| Final INY KO Assembly R |  | TCGCTAAAACCAGACCAATT |  |  |  |
| Final INY KO Assembly F | NVM025S01 and NVM076S08 INY KO Gibson assemblies | ACAATGGCTGCTGATCGTGG | Primers used to amplify finalized Gibson assemblies | | Gibson assembly of inerolysin knockout PCR products |
| Final INY KO Assembly R |  | CGGGGTTTTTATAGCGTGCG |  |  |  |
| Recipient F | Tested on NVM196S18 and NVM041S27 wildtype and NVM196S18 gDNA transformant colonies | TCATTGGGCTCATTTTTCTTCGC | Primers used for identifying the NVM196S18-specific gene (N^6^-methyltransferase) | | PCR identification of gDNA transformants and PCR knockout transformants |
| Recipient R |  | TGTACGAACATACCTCCAGAACC |  |  |  |
| ermB ID F | Tested on NVM196S18 and NVM041S27 wildtype and NVM196S18 gDNA transformant colonies | CTGAATCGAGACTTGAGTGTGC | Primers used for identifying the *ermB* cassette | | PCR identification of gDNA transformants and PCR knockout transformants |
| ermB ID R |  | GGGCATTTAACGACGAAACTGG |  |  |  |
| Donor F | Tested on NVM196S18 and NVM041S27 wildtype and NVM196S18 gDNA transformant colonies | GTCAATGATGTCACAAGCCCG | Primers used for identifying the NVM041S27-specific gene (Cas9) | | PCR identification of gDNA transformants and PCR knockout transformants |
| Donor F |  | ATTCCTGTCACTATAACATCGCC |  |  |  |
| INY KO Insertion F | Tested on NVM196S18 wildtype and NVM196S18 INY KO transformant colonies | TGCTCATCCAACAACTAGCT | Primers flanking the middle of the inerolysin gene to confirm the *ermBL* insertion occurred | | PCR identification of gDNA transformants and PCR knockout transformants |
| INY KO Insertion R |  | CTGTTGCATCCCCAGAGTTA |  |  |  |
| INY KO Integration F | Tested on NVM196S18 wildtype and NVM196S18 INY KO transformant colonies | GTCTGCCTATGGTAAGTTGGGA | Primers flanking the 5’ end of the PCR cassette and one annealing to ermB to confirm genomic insertion | | PCR identification of gDNA transformants and PCR knockout transformants |
| INY KO Integration R |  | AGCAATGAAACACGCCAAAGT |  |  |  |
| comGA Upstream F | NVM076S08 gDNA | TAGCCAATTTGACTTATGATGTTGTTGA | Amplifies first half and upstream region (total length of 2 kb) of NVM076S08 comGA for Gibson assembly. comGA Upstream F also used to amplify entire fragment. | | Gibson assembly of *comGA* knockout PCR products |
| comGA Upstream R |  | TAATAATGTACCAATATTTGATCTTCAATCGTCATAACAAATTTTT |  |  |  |
| tetM F | NVM041S27 gDNA (has overhangs with homology to NVM076S08 comGA) | TTGTTATGACGATTGAAGATCAAATATTGGTACATTATTACAGCTAT | Amplifies tetM from NVM041S27 for use in comGA knockout Gibson assembly. | |  |
| tetM R |  | GTTCTAATATTTCAATTGGAAATAACTATCTCCTCCTTTACACT |  |  |  |
| comGA Downstream F | NVM076S08 gDNA | TAAAGGAGGAGATAGTTATTTCCAATTGAAATATTAGAACCTGATT | Amplifies second half and downstream region (total length of 2 kb) of NVM076S08 comGA for Gibson assembly. comGA Downstream R also used to amplify entire fragment. | |  |
| comGA Downstream R |  | AAATATACCCGTTTATTGATAATTGACTCTTTCA |  |  |  |
| comGA Integration F | Tested on NVM076S08 and NVM041S27 wildtype and NVM076S08 comGA KO transformant colonies | GCGGATTTATTACAACACTGATGGC | Primers to confirm insertion of comGA knockout fragment into NVM076S08 genome. Forward targets NVM076S08 genome upstream of insertion fragment, reverse targets *tetM*. | | PCR identification of PCR knockout transformants |
| comGA Integration R |  | ATGATGTTCACCTTCGTATTTTCCC |  |  |  |

**Table S3. Predicted methylation motifs in Nanopore-sequenced *L. iners* strains.** Methylation sites were identified from Nanopore sequencing data using Modkit. The Modification column indicates the type of base modification: 5mC (5-methylcytosine) or 6mA (N6-methyladenine). In the Motif column, brackets [] indicate the methylated position. For each motif, Modkit calculates a per-site fraction modified, defined as N_mod / (N_canonical + N_mod + N_other_mod), where N_mod is the number of base calls passing modification filters, N_canonical is the number not passing modification filters, and N_other_mod is the number passing filters for a different modification of the same base. Each genomic site matching a given motif is then classified based on this fraction: High (>= 0.6), Low (< 0.2), or Medium (between 0.2 and 0.6). The High Count, Low Count, and Mid Count columns report the number of genomic sites for that motif falling into each category. The High Count / (High Count + Low Count) column gives the proportion of classifiable sites that are in the High category. The log2odds column is the log2 of the odds of a site being classified as High rather than not High.

| ***L. iners***  **Strain** | **Modification** | **Motif** | **High Count / (High Count + Low Count)** | **High Count** | **Low Count** | **Mid Count** | **log_2_odds** |
| --- | --- | --- | --- | --- | --- | --- | --- |
| **NVM020S14** | 5mC | G[C]GC | 0.995169 | 206 | 1 | 7 | infinite |
|  | 6mA | GCCA[A]T | 0.994758 | 759 | 4 | 59 | 18.23566 |
|  | 6mA | RCA[A]NNNNNRTAC | 0.944444 | 136 | 8 | 43 | 13.65544 |
|  | 6mA | CT[A]NNNNNGTC | 0.925134 | 173 | 14 | 71 | 13.24169 |
|  | 6mA | GT[A]YNNNNNTTGT | 0.868852 | 53 | 8 | 38 | 12.19686 |
|  | 6mA | G[A]CNNNNNTAG | 0.722222 | 117 | 45 | 102 | 10.92315 |
| **NVM025S01** | 5mC | G[C]GC | 1 | 199 | 0 | 0 | infinite |
|  | 6mA | CG[A]GC | 0.9838129 | 547 | 9 | 92 | 14.562123 |
|  | 6mA | CCC[A]T | 0.9755725 | 1278 | 32 | 173 | 14.516685 |
|  | 6mA | C[A]YNNNNNNNTYTC | 0.9707602 | 498 | 15 | 76 | 13.658957 |
|  | 6mA | GAR[A]NNNNNNNRTG | 0.9484127 | 478 | 26 | 84 | 12.793871 |
| **NVM041S27** | 5mC | G[C]GC | 0.984127 | 186 | 3 | 11 | 15.32353 |
|  | 5mC | GGNN[C]C | 0.726467 | 656 | 247 | 386 | 12.59611 |
| **NVM076S08** | 5mC | G[C]GC | 1 | 116 | 0 | 3 | infinite |
|  | 6mA | CCC[A]T | 0.933457 | 505 | 36 | 176 | 13.08927 |
|  | 6mA | GT[A]NYNNNNNTCNNC | 0.927273 | 51 | 4 | 16 | 12.37289 |
|  | 6mA | CG[A]GC | 0.902778 | 195 | 21 | 79 | 12.07515 |
|  | 6mA | CT[A]NNNNNGTC | 0.880597 | 59 | 8 | 29 | 11.59152 |
|  | 6mA | CTGC[A]G | 0.809524 | 187 | 44 | 130 | 10.93819 |
| **NVM196S18** | 5mC | G[C]GC | 1 | 218 | 0 | 0 | inf |
|  | 6mA | G[A]CNNNNNTAG | 1 | 296 | 0 | 2 | inf |
|  | 6mA | T[A]YCNNNNTCT | 1 | 592 | 0 | 1 | inf |
|  | 6mA | GA[A]NNNNNNTTYG | 1 | 541 | 0 | 0 | inf |
|  | 6mA | CT[A]NNNNNGTC | 1 | 298 | 0 | 0 | inf |
|  | 6mA | CRA[A]NNNNNNTTC | 1 | 541 | 0 | 0 | inf |
|  | 6mA | [A]GANNNNGRTA | 0.9982877 | 583 | 1 | 7 | 17.42559 |
| **NVM210S01** | 5mC | G[C]GC | 1 | 195 | 0 | 9 | infinite |
|  | 6mA | GC[A]NNNNNGTG | 0.97351 | 147 | 4 | 75 | 15.98261 |
|  | 6mA | C[A]CNNNNNTGC | 0.967949 | 151 | 5 | 71 | 15.71073 |
|  | 6mA | GAGG[A] | 0.579661 | 342 | 248 | 568 | 11.93787 |
